# Gut Microbiota Is Linked to Female Olfactory Preference in Giant Pandas

**DOI:** 10.3390/ani16162574

**Published:** 2026-08-18

**Authors:** Yan He, Xiaoyan Liu, Kexin Xu, Zheng Yan, Rongping Wei, Mingyue Zhang, Guiquan Zhang, Dingzhen Liu

**Affiliations:** 1 Key Laboratory of Biodiversity Science and Ecological Engineering of Ministry of Education, Department of Ecology, College of Life Sciences, Beijing Normal University, Beijing 100875, China; 202331200049@mail.bnu.edu.cn (Y.H.);; 2 Key Laboratory of State Forestry and Grassland Administration on Conservation Biology of Rare Animals in the Giant Panda National Park, China Conservation and Research Center for the Giant Panda, Chengdu 611830, China; 3 The Conservation of Endangered Wildlife Key Laboratory of Sichuan Province, Chengdu Research Base of Giant Panda Breeding, Chengdu 611830, China; zmy6611@126.com

**Keywords:** giant panda, exploration preference, gut microbiota, olfactory preference

## Abstract

Mate choice can vary with internal condition, but whether gut microbial variation is associated with female mate-choice-related olfactory preferences remains poorly understood. In this study, we tested whether dietary dried yeast supplementation was associated with changes in gut microbiota and odor preference in captive female giant pandas (*Ailuropoda melanoleuca*). Females in the dried yeast supplementation group received 10 g of dried yeast tablets daily for five consecutive days, while control females received the same diet without supplementation. Female olfactory preferences for urine from adult and senile males were assessed using odor preference tests before and after supplementation, and fecal samples were analyzed to characterize gut bacterial communities. Dried yeast supplementation altered the relative abundances of several gut bacterial taxa. Putatively beneficial taxa, including Muribaculaceae and *Weissella cibaria*, increased, whereas potentially pathogenic or opportunistic taxa, including Enterobacteriaceae and *Campylobacter*, decreased (all adjusted *p* < 0.05). Before supplementation, females showed no significant preference between adult and senile male urine. After supplementation, females showed a significant preference for adult male urine. Several gut bacterial genera were significantly associated with female exploratory behavior. These results suggest a possible association between dried yeast supplementation, gut microbial shifts, and female olfactory responses to male urinary cues.

## Simple Summary

Mate choice can affect breeding success in animals, but the factors that influence female preference are not fully understood. Gut bacteria are increasingly recognized as important for animal health and behavior. In this study, we tested whether changing the gut bacteria of captive female giant pandas through dried yeast tablet supplementation was associated with changes in their responses to male urine odors. Female giant pandas were presented with urine from adult and senile males before and after supplementation. We also compared their gut bacterial communities before and after treatment. After dried yeast supplementation, several potentially beneficial gut bacteria increased, whereas some potentially harmful bacteria decreased. Before supplementation, females did not show a clear preference between adult and senile male urine. After supplementation, however, females showed a stronger preference for adult male urine. These results suggest that dietary supplementation may be associated with changes in gut bacteria and female odor-based mate-choice responses in giant pandas. This study may provide useful information for improving reproductive management in captive breeding programs.

## 1. Introduction

Sexual selection theory predicts that females are expected to be more selective during mate choice due to the fact that they bear greater reproductive investment and parental-care costs than males [1,2]. Females integrate multimodal signals, including visual, olfactory, and acoustic cues, to evaluate male quality and preferentially mate with males that may enhance their offspring fitness [3,4,5]. Among these signals, urine and anogenital gland secretions are key chemical carriers that can encode individual information, including sex, age, reproductive status, and genetic background [6,7,8], and thus can play an important role in female mate choice and sexual selection. From the perspectives of sexual and natural selection, prime-aged adult males are expected to be more attractive than subadult or senile males because they have reached reproductive maturity, may possess stronger competitive ability, and are less likely to show age-related declines in ejaculate quality, fertilization capacity, and mating performance [9,10,11]. Choosing prime-aged adult males may increase the probability of successful reproduction and potentially enhance offspring fitness [11,12,13]. Age-related odor cues may therefore be especially relevant to female mate assessment [9,14]. However, female mating preferences are not fixed but shaped by male traits, female physiological state [15,16], male quality [17,18], and microbiota [19,20].

Gut microbiota regulates host metabolism, and behavior through the microbiota–gut–brain axis (MGBA) [21,22], and they have co-evolved with their hosts through reciprocal and often mutualistic interactions [23,24]. In humans, livestock, and captive animals, antibiotics and probiotic or prebiotic preparations are widely used to modify gut microbiota, with the aim of improving animals’ physiological state [25,26]. In recent years, studies of microbe–host interactions have frequently used antibiotics to induce gut microbial dysbiosis in model organisms, including mice and zebrafish [27,28]. This approach has helped reveal microbial contributions to host behavioral responses, and cognition regulation [27,28,29]. Accumulating evidence also suggests that gut microbiota can influence mate choice directly or indirectly by modulating pheromone or odor profiles [30], host behavioral responsiveness [22,31] and intestinal health [19,32,33]. However, most evidence comes from insects and laboratory rodents, and whether gut microbial modulation affects mate-choice-related olfactory preferences in large territorial mammals remains largely unknown [34].

The giant panda is a vulnerable and flagship species endemic to China. Giant pandas exhibit highly seasonal reproduction, with females experiencing a brief annual estrous period that restricts opportunities for successful mating [35,36]. As solitary mammals, giant pandas rely heavily on chemical communication [37,38,39]. During mating season, both wild and captive giant pandas show pronounced estrous and mate-searching behaviors [39,40,41]. Therefore, efficient mate assessment and reproductive communication are critical for breeding success, particularly in captive conservation programs [42,43]. Previous studies in captive populations indicate that female mate choice and mate preference can improve copulation success and birth rates [13,15]. The captive population has expanded steadily through sustained management efforts since 1999 [44]. In practice, senile males in captivity are excluded from the annual breeding program due to their low attractiveness to female, poor sexual performance and reproductive potential. Despite retaining a gastrointestinal anatomy typical of carnivores, giant pandas have evolved a highly specialized diet composed almost entirely of fibrous bamboo, making them a distinctive model for studying diet–microbiome interactions [45]. Although their capacity for fiber degradation appears limited compared with that of specialized herbivores, gut microorganisms may contribute to the processing of plant-derived polysaccharides and the production of microbial metabolites [46,47,48]. In this context, variation in gut microbial composition likely reflects complex interactions among dietary inputs, microbial ecology, and host physiological status [45,46,47,48]. Our previous work showed that gut microbiota composition and function are associated with natural mating ability in male giant pandas [49]. However, whether dietary modulation of the gut microbiota is associated with behavioral traits involved in social and reproductive communication remains largely unexplored [49,50].

An increasing body of evidence indicates that female mate choice is condition-dependent, such that females in better condition tend to exhibit proper and stronger mating preferences [51]. Therefore, we hypothesized that changes in gut microbial community structure would be associated with changes in female olfactory discrimination of male urinary odors and stronger preferences for chemosensory cues from adult males. To test this hypothesis, we provided dietary dried yeast supplementation to captive female giant pandas and examined whether this treatment was associated with changes in their gut microbiota and olfactory preferences for urine from males of different age classes. Moreover, emerging evidence links gut microbial composition to male mating performance in this species. Together, these characteristics provide an opportunity to examine whether variation in the female gut microbiota is associated with mate-choice-related olfactory responses in a conservation-relevant mammal. Our findings may provide both mechanistic insight into microbial contributions to sexual selection and practical guidance for optimizing mate-pairing strategies, improving natural mating efficiency, and supporting the sustainable management of captive giant panda populations.

## 2. Materials and Methods

### 2.1. Study Animals and Housing Conditions

This study was conducted from 10 March to 23 May 2023. A total of 27 healthy giant pandas (12 males and 10 females) in the China Conservation and Research Center for the Giant Panda (CCRCGP) were selected as research subjects (Table S1). Fifteen females were randomly assigned to either the dried yeast-supplemented treated group (DS-treated group, *n* = 5) administered dried yeast tablets or the control group (*n* = 5) that received no additional treatment. In addition, twelve male pandas were divided into adult group (*n* = 6) and senile group (*n* = 6) by referring to the age group classification criteria for giant pandas [52]. Those males were used as urine donors for subsequent odor preference test.

**Figure 1:**
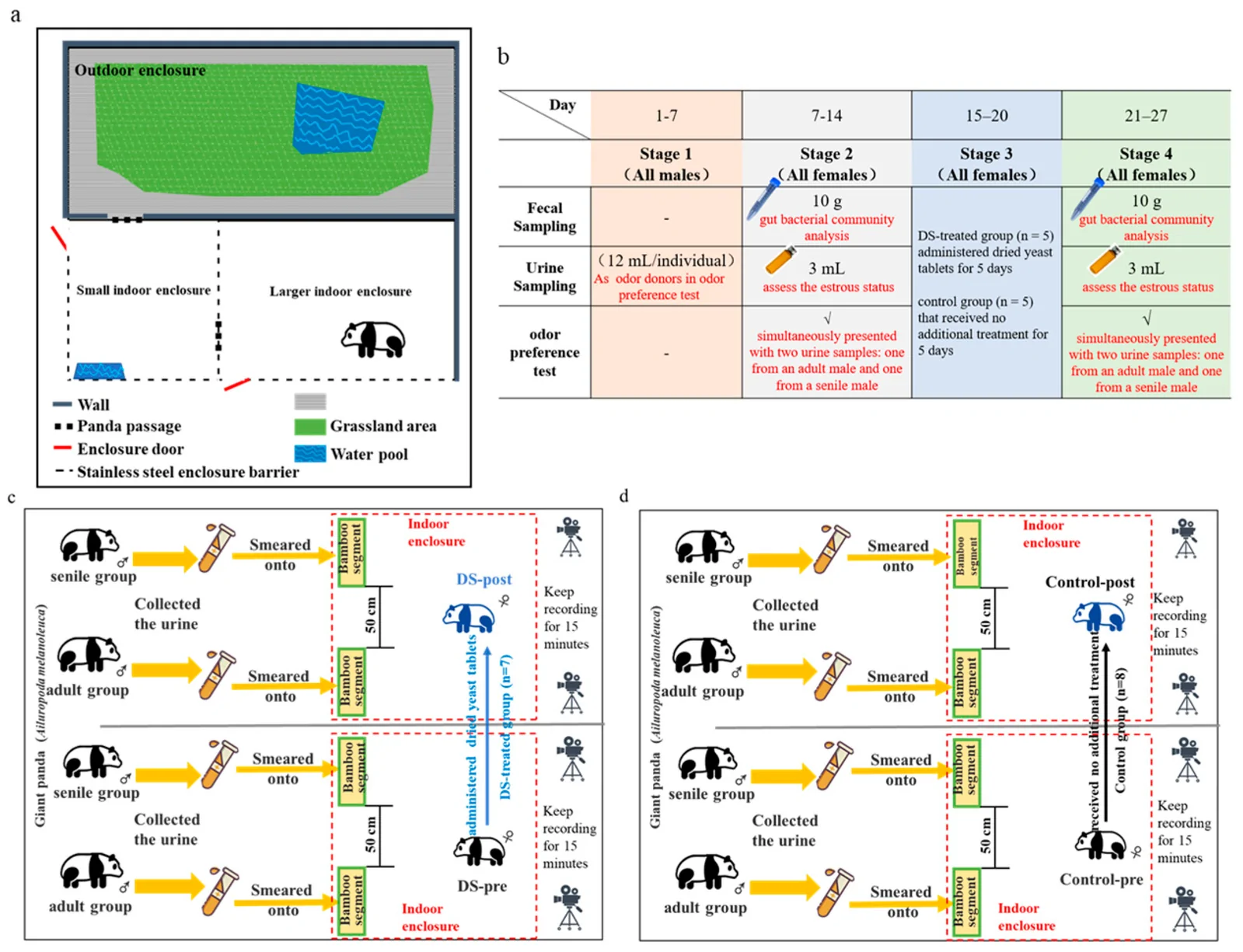
Schematic overview of the study design. (**a**) Housing layout, (**b**) experimental design, and odor preference test procedure in female giant pandas (**c**,**d**).

All subjects were housed individually, with their activity areas comprising an outdoor enclosure of approximately 200 m^2^ and two indoor enclosures of each approximately 10 m^2^ (Figure 1a). Panda keepers performed daily routine cleaning of the subjects’ activity areas from 8:35 a.m. to 10:00 a.m. The center provided an ample daily supply of bamboo. In addition, each panda was provided with an appropriate quantity of bamboo shoots and Wotou twice daily. The Wotou were mainly composed of corn, soybeans, rice, sugar, eggs, K_2_HPO_4_, salt, and vegetable oil. The amounts of bamboo shoots and Wotou were determined based on the body weight and physical condition of each panda.

### 2.2. Dietary Dried Yeast Tablet Supplementation and Sample Collection

*Dietary dried yeast tablet supplementation*. In accordance with routine health-management practices at the CCRCGP and veterinary recommendations, dried yeast tablets (Guangdong Wuzhou Pharmaceuticals, Zhanjiang, China) were used as a dietary supplement in this study. During the supplementation period, each female in the DS-treated group received 10 g of dried yeast tablets daily for five consecutive days (Figure 1b). The dosage was selected based on the manufacturer’s instructions and veterinary advice. The tablets were ground into powder and thoroughly mixed with the routinely provided Wotou before feeding. Females in the control group received the same Wotou without dried yeast powder. All other dietary, housing, and husbandry conditions were kept consistent between the two groups.

*Male urine collection for odor preference tests*. Urine samples used as odor stimuli were collected from adult and senile male odor donors within one week before the formal odor preference tests (Table S1, Figure 1b). All male urine samples were collected following Liu et al. (2008), immediately transferred into sterile centrifuge tubes, and stored at −80 °C until use [8].

*Female urine collection for estrous-state assessment*. Before each odor preference test, the estrous state of each female was assessed using both behavioral observations and urinary hormone measurements [53]. Female urine samples from both the DS and control groups were collected on the day preceding each odor preference test. Urinary estrone-3-glucuronide (E1G) concentrations were measured by enzyme-linked immunosorbent assay (ELISA) and used as an indicator of female estrous status.

*Fecal sample collection for gut microbiota analysis*. Fresh fecal samples were collected from female giant pandas on the day of each odor preference test, immediately after defecation, following the protocol described by Yan et al. (2024) [49]. Samples were transferred into sterile tubes and stored at −80 °C until further analysis. These fecal samples were used to characterize the gut bacterial communities of females before and after dried yeast tablet supplementation.

### 2.3. Urine Odor Preference Tests

Each female in the DS and control groups underwent two odor preference tests, i.e., one before and one after the five-day dietary supplementation experimental period, respectively. Odor preference tests were conducted from 10:00 to 11:30 or 14:00 to 15:00 during which the pandas were active. All tests were performed in an indoor enclosure to minimize external disturbance. On the day of each odor preference test, frozen male urine samples were thawed at room temperature before presentation. In the test, each female was exposed synchronously to two urine samples from one adult and one senile male. For each female, the urine samples used in the pre- and post-treatment odor preference tests were obtained from the same adult male and the same senescent male, which were randomly selected from candidate males with a pairwise male–female relatedness coefficient of γ < 0.125 (to minimize potential effects of kinship). The left–right position of the two urine stimuli was randomly assigned in each test, and the urine volume was standardized at 2.5 mL for each stimulus. On the day of each odor preference test, the estrous state of each female was assessed using both behavioral observations and urinary hormone measurements [53].

Two video cameras (HDR-CX610E; Sony, Shanghai, China) were used to record the female’s behavior (Figure 1c,d). Recording began when all four limbs of the female had entered the larger indoor enclosure from the small one and continued for 15 min (Figure 1). Females’ preference behaviors were recorded using focal-animal sampling and continuous recording [54]. Based on established ethograms for giant pandas, exploratory behaviors, including sniffing and licking [41,55], were classified as either non-contact or contact exploration (Table S2). Three trained observers coded the behavior video recordings using a single-blind procedure. For each urine stimulus, the observers recorded exploration frequency, total exploration duration in seconds. The latency of the first-time exploration to the stimulus was defined as the interval between the female’s entry into the large indoor enclosure and her first exploratory response to the stimulus. Overall inter-observer agreement exceeded 92%.

To quantify females’ exploratory preferences for urine from males of different age classes, an exploration preference index was calculated as follows [56,57]: *Pi*_exploration = (t__adult_ − t__senile_)/(t__adult_ + t_s_enile_). Here, t__adult_ represents the duration of exploring urine from adult males, and t__senile_ denotes the duration of exploring urine from senile males. This index ranges from −1 to +1, with negative value indicates a preference for urine from senile males, while a positive value indicates a preference for urine from adult males. A one-sample *t*-test was used to examine whether the preference index significantly differed from zero. Cohen’s d was calculated as the mean preference index divided by its standard deviation to quantify the standardized difference from zero; an absolute value of *d* > 1.0 was considered a large effect.

### 2.4. 16S rRNA Gene Amplicon Sequencing and Analysis of Fecal Samples

The total DNA was extracted from female fecal samples using a DNA extraction kit (TianGen Biotech Co., Ltd., Beijing, China) according to the manufacturer’s instructions. The bacterial 16S rRNA gene was amplified using the primer pairs F515 and R806 [58]. Sequencing libraries were constructed following the manufacturer’s protocol and subsequently quantified using Qubit and quantitative-PCR. Qualified libraries were sequenced on an Illumina NovaSeq 6000 platform (Illumina, San Diego, CA, USA). Raw sequencing data were processed using QIIME 2 (Caporaso Lab, Arizona, USA) following previously described pipelines [59]. The resulting sequence data were for downstream analyses of bacterial diversity, taxonomic composition, and differential abundance between pre- and post-treatment.

### 2.5. Data Analysis

Urinary E1G concentrations did not differ significantly among females before the behavioral tests, suggesting comparable reproductive status across individuals. Therefore, E1G was not included as a covariate in the main behavioral analyses. To confirm the robustness of the result, we further performed sensitivity analysis including E1G as a covariate, which yielded qualitatively similar result.

The Shapiro–Wilk test was used to assess the normality of continuous variables, including gut bacterial α-diversity indices and female behavioral metrics, such as sniffing frequency, duration, and latency. For paired comparisons, paired *t*-tests or Wilcoxon signed-rank tests were used as appropriate. These included comparisons of female responses to adult versus senescent male urine within the same odor preference test, as well as comparisons of female responses to the same odor type before and after dried yeast supplementation. For comparisons between independent groups, such as DS-treated and control females, independent-samples *t*-tests or Mann–Whitney U tests were used depending on data normality. When more than two independent groups were compared, global differences were assessed using the Kruskal–Wallis H test, followed by Dunn’s post hoc tests for pairwise comparisons. For multiple comparisons, raw *p* values were adjusted using the Benjamini–Hochberg false discovery rate (FDR) method, and FDR-corrected *p* values were reported where applicable.

Differential abundance analysis of bacterial taxa between pre- and post-supplementation samples was performed using edgeR, and *p* values were adjusted using the Benjamini–Hochberg FDR method. Correlation analysis was conducted using the psych package (2.6.1) in R (version 20260101, RStudio PBC, Boston, MA, USA), with *p* values adjusted using the Benjamini–Hochberg FDR method.

## 3. Results

### 3.1. Dried Yeast Supplementation Altered the Relative Abundances of Specific Gut Bacterial Taxa

A total of 2,589,143 ASVs were obtained from 20 fecal samples of female giant pandas by 16S rRNA gene amplicon sequencing, with an average of 116,294 sequences per sample. No significant differences in bacterial α-diversity indices were detected among the four sampling groups (DS–pre, DS–post, control–pre, and control–post; Dunn’s test, *p* > 0.05) (Figure S1). Ordination analysis of β-diversity revealed no clear separation between pre- and post-treatment samples in either the DS or the control group (Figure S2). In this study, Firmicutes and Proteobacteria were the dominant phyla in the gut microbiota of females, together accounting for more than 95% of the total relative abundance (Figure S3a). The dominant genera included *Streptococcus*, *Clostridium*, *Turicibacter*, and *Escherichia-Shigella* (Figure S3b).

**Figure 2:**
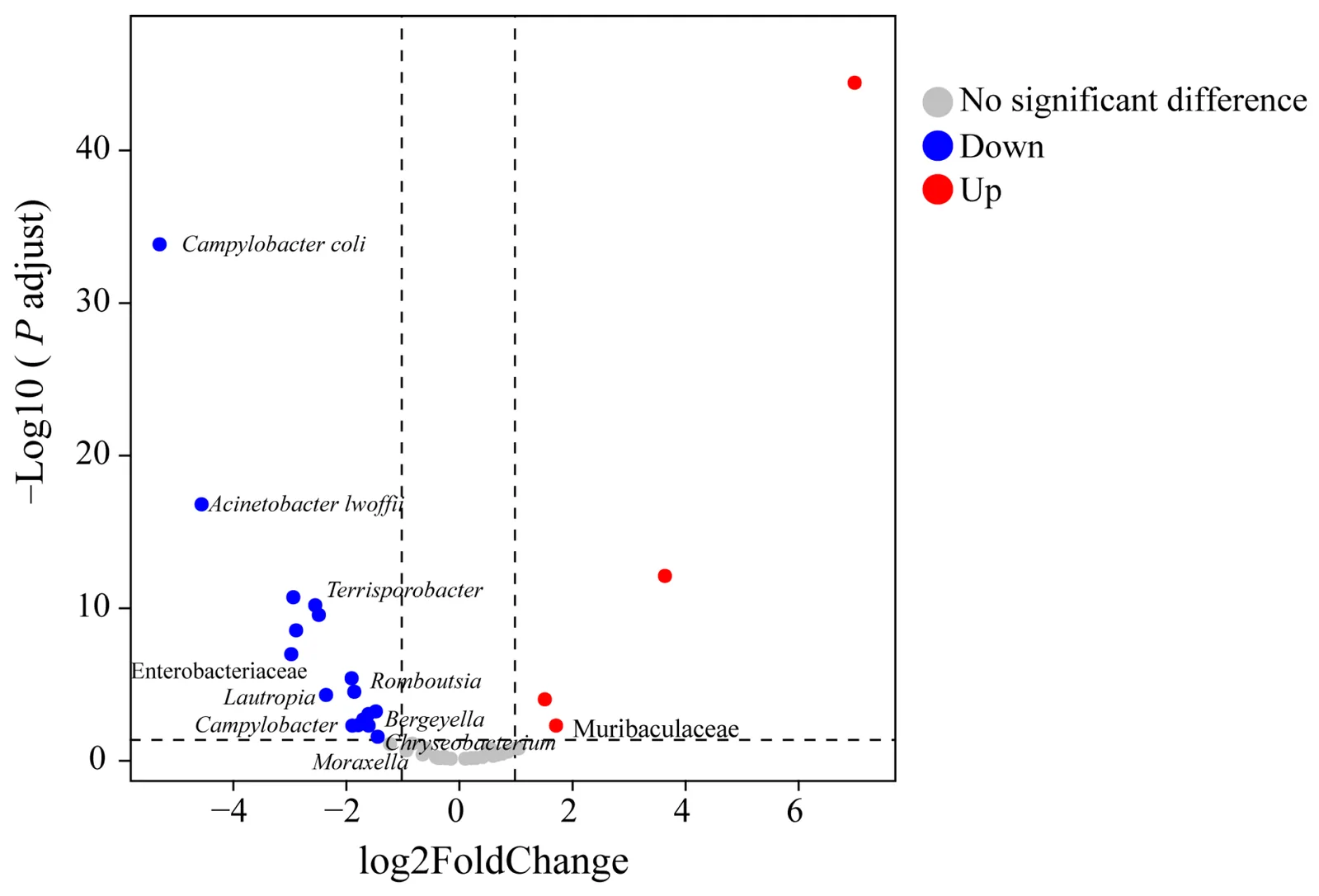
Volcano plot showing differences in gut bacterial taxa between DS–pre and DS–post samples based on edgeR analysis. Red points indicate taxa significantly increased after dried yeast supplementation, blue points taxa significantly decreased after dried yeast supplementation, and gray points indicate taxa with no significant difference. The vertical dashed lines indicate the log2 fold-change thresholds, and the horizontal dashed line indicates the statistical significance threshold based on the adjusted *p* value.

No bacterial taxa differed significantly between the pre- and post-treatment samples in the control group (*P_adjust_* > 0.05). In contrast, DS–pre and DS–post samples differed significantly in 19 bacterial taxa (*P_adjust_* < 0.05). Specifically, the relative abundances of genera were significantly lower in DS–post than in DS–pre, including *Campylobacter* (*P_adjust_* = 0.006), *Terrisporobacter* (*P_adjust_* < 0.001), *Lautropia* (*P_adjust_* < 0.001), *Romboutsia* (*P_adjust_* < 0.001), *Moraxella* (*P_adjust_* = 0.002), *Bergeyella* (*P_adjust_* = 0.001), *Chryseobacterium* (*P_adjust_* = 0.006), *Actinomyces* (*P_adjust_* = 0.0007), and *Leucobacter* (*P_adjust_* = 0.03) (Figure 2). The relative abundances of *Weissella cibaria*, Muribaculaceae, and *Rothia endophytica* were significantly higher after dried yeast supplementation (*P_adjust_* < 0.001).

### 3.2. Dietary Supplementation with Dried Yeast Tablets Significantly Altered Olfactory Preferences in Female Giant Pandas

**Figure 3:**
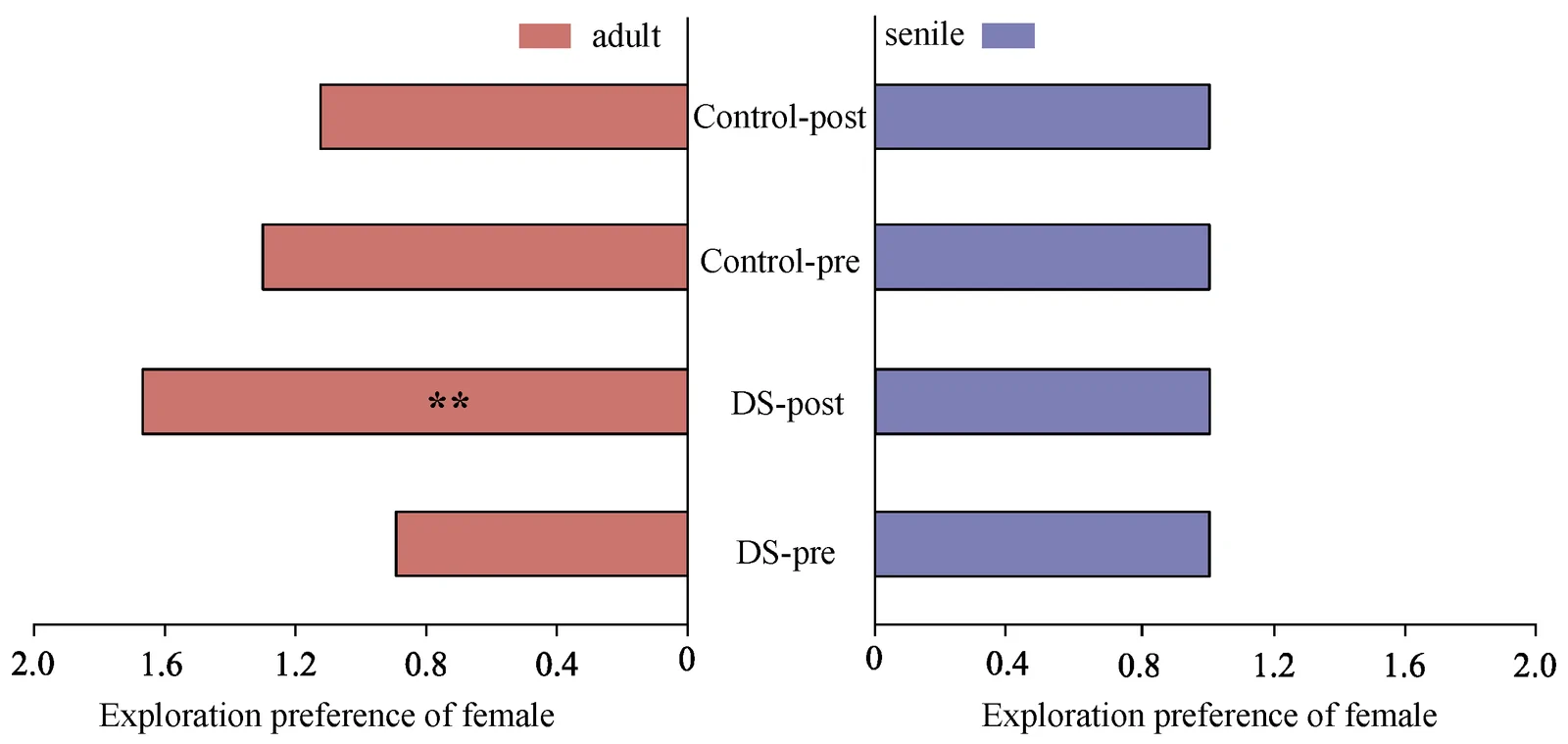
Exploration preference index of female giant pandas toward urine from adult and senile males in the odor preference test. ** *p* < 0.01.

**Figure 4:**
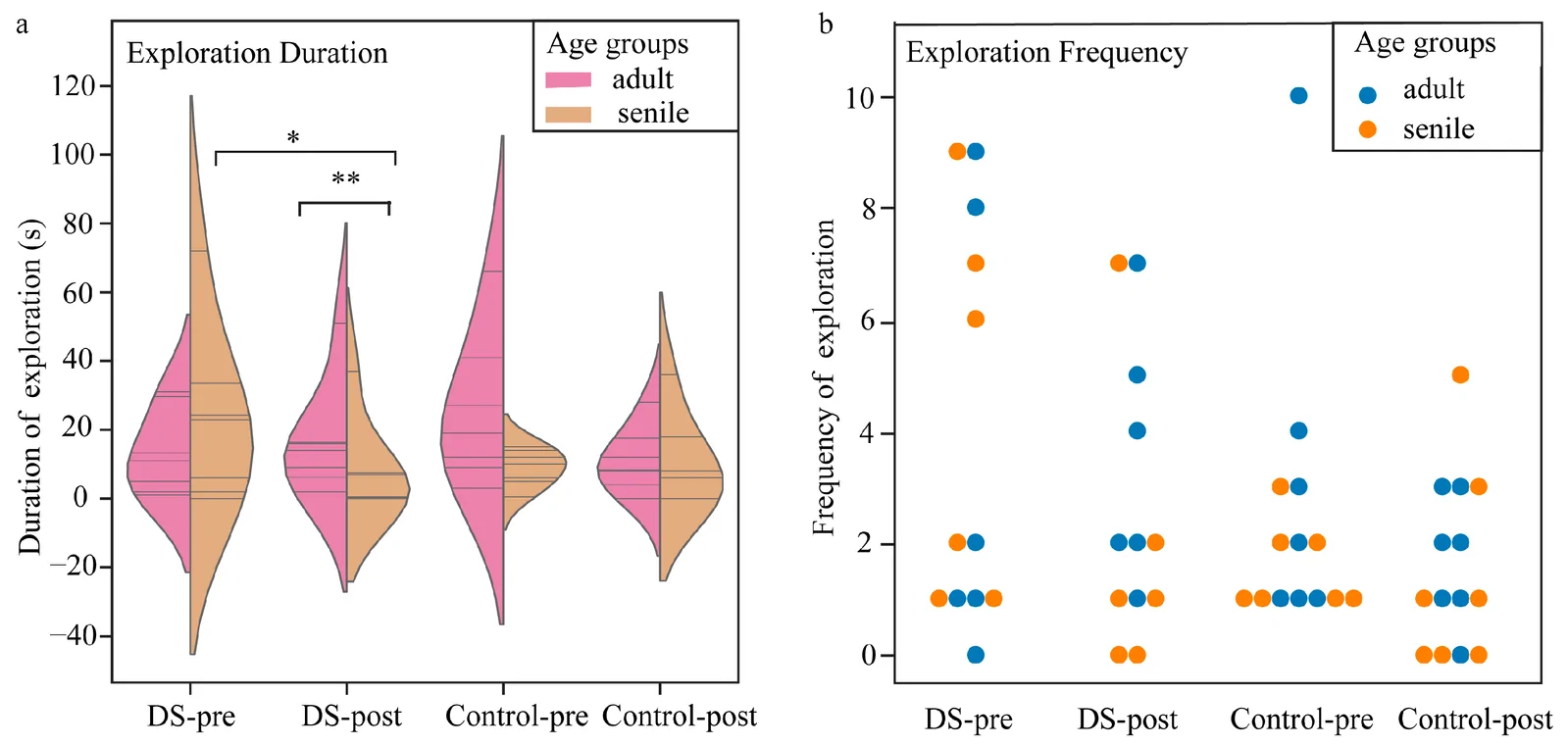
Exploration duration (**a**) and exploration frequency (**b**) of female giant panda in response to urine from adult and senile males in the odor preference test. ** *p* < 0.01, * *p* < 0.05.

**Figure 5:**
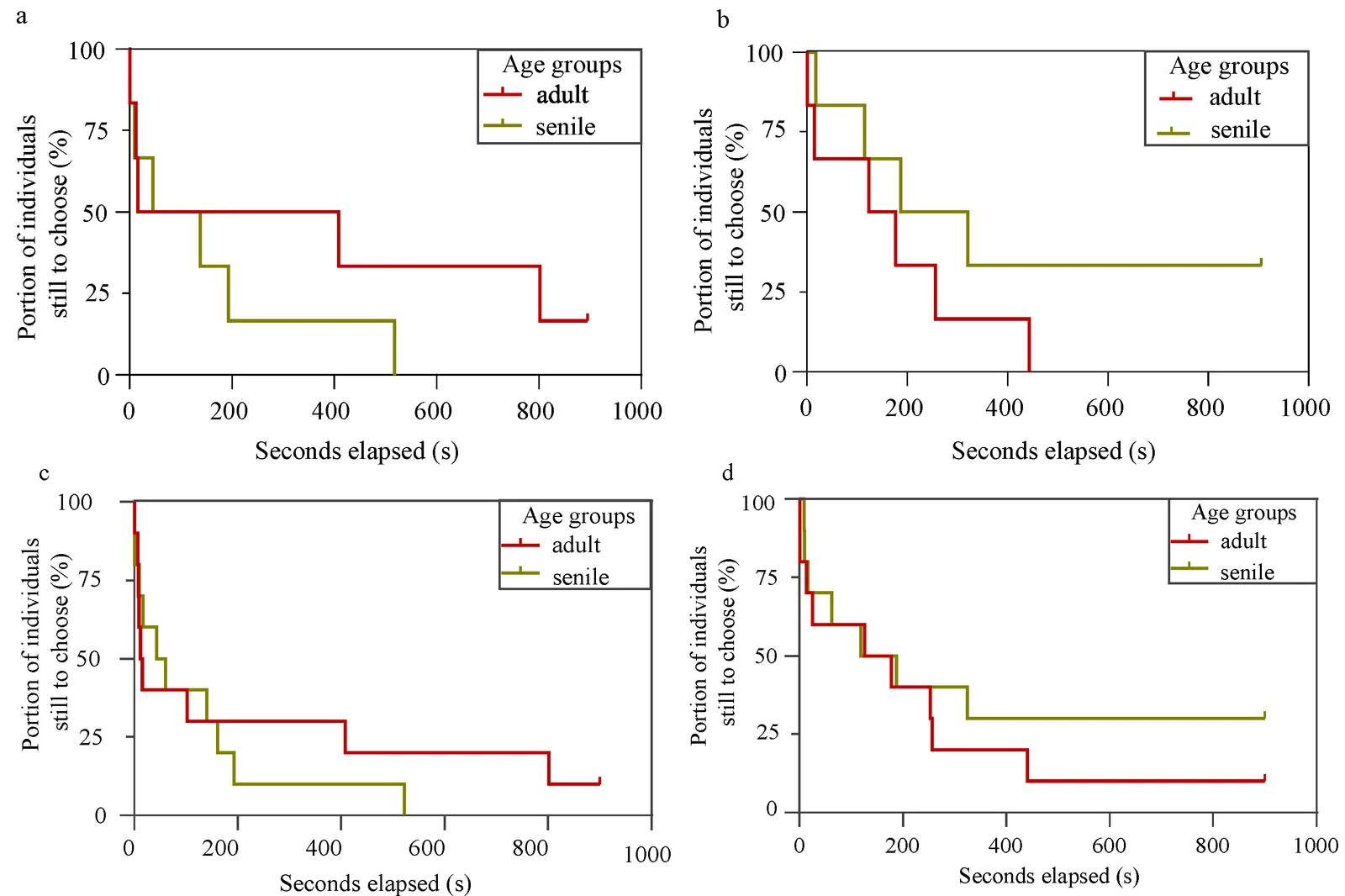
Survival curves showing the latency to first exploration of urine from adult and senile male giant pandas in the odor preference test. Survival curves were compared using log-rank test (Mantel–Cox test). Panels show female responses in different experimental groups: (**a**) DS–pre group; (**b**) DS–post group (**c**); control–pre group; (**d**) control–post group.

The exploration preference index (*Pi*_exploration) revealed that females in the DS-treated group exhibited a significant preference for urine from adult males after dried yeast tablets supplementation (one-sample *t*-test: *t* = 4.500, *p* = 0.006, 95% CI: 0.288–1.055, Cohen’s d = 1.84) (Figure 3). Before supplementation, the DS-treated group showed no olfactory preference for urine from either adult or senile males (all *p* > 0.05) (Figure 4a,b). After supplementation, the exploration duration of the DS-treated group toward urine from adult males was significantly longer than that toward urine from senile males (*t* = 4.089, *p* = 0.009) (Figure 4a). Compared with DS–pre, DS–post females displayed significantly decreased exploration frequency (*t* = 2.858, *p* = 0.0369) and duration (*t* = 2.878, *p* = 0.0358) toward urine from senile males (Figure 4a,b). After supplementation, DS–post females exhibited a shortened latency to first exploration of the urine of adult males and an increased latency to first exploration of the urine odor of senile males (Figure 5a,b).

Females in the control group showed no preference for urine from males of different age groups (Figure 3), with no significant changes in exploration frequency or duration (all *p* > 0.05) (Figure 4a,b). In odor preference test, females from pre- and post-control groups showed no significant differences in latency to first investigation of male urine from different age groups (Figure 5c,d).

### 3.3. Correlation of Gut Microbiota with Exploratory Behavior in Female Giant Pandas

**Figure 6:**
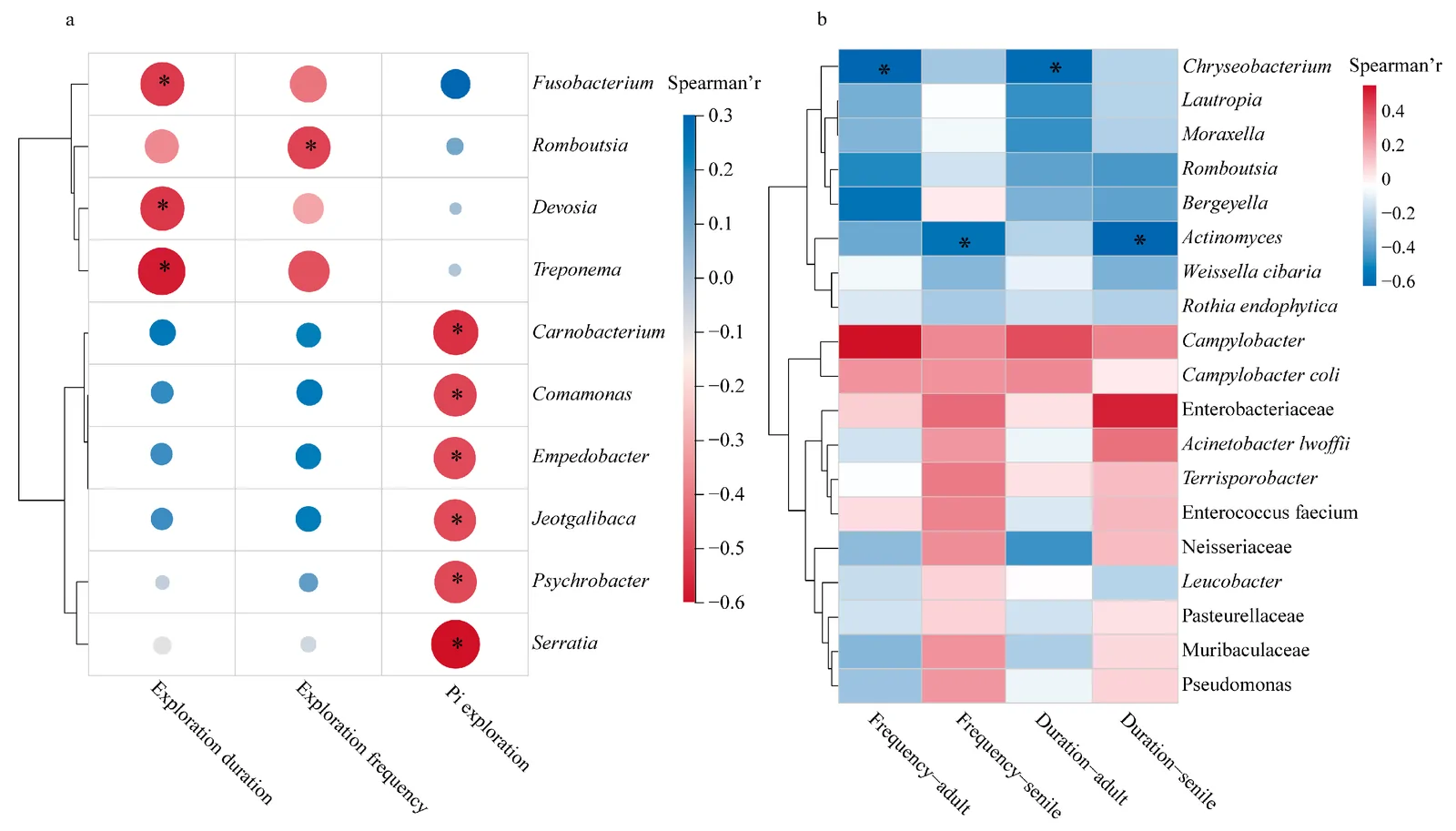
Spearman correlation analysis between gut bacterial taxa and female exploration behavior metrics in the odor preference test. (**a**) Correlations between the top 100 most abundant gut bacterial genera and exploration behavior metrics. (**b**) Correlations between differentially abundant gut bacterial taxa identified by edgeR and exploration behavior metrics. * *P_adjust_* < 0.05.

Spearman correlation analysis revealed that the relative abundances of gut bacterial genera were significantly correlated with exploration behavior in female giant pandas. Specifically, the abundances of *Treponema* (*r* = −0.56, *P_adjust_* = 0.016), *Fusobacterium* (*r* = −0.51, *P_adjust_* = 0.032), and *Devosia* (*r* = −0.51, *P_adjust_* = 0.031) were significantly negatively correlated with exploration duration, while the relative abundance of *Romboutsia* (*r* = −0.49, *P_adjust_* = 0.040) was significantly negatively correlated with the exploration frequency (Figure 6a). In addition, the relative abundances of *Serratia* (*r* = −0.58, *P_adjust_* = 0.011), *Carnobacterium* (*r* = −0.52, *P_adjust_* = 0.026), *Comamonas* (*r* = −0.49, *P_adjust_* = 0.040), *Psychrobacter* (*r* = −0.48, *P_adjust_* = 0.042), *Empedobacter* (*r* = −0.48, *P_adjust_* = 0.046), and *Jeotgalibaca* (*r* = −0.48, *P_adjust_* = 0.046) were significantly negatively correlated with the exploration preference index (*Pi*_exploration) (Figure 6a). Among the 19 differentially abundant bacterial taxa identified between DS–pre and DS–post, and the relative abundance of *Actinomyces* was significantly negatively correlated with both the frequency (*r* = −0.68, *P_adjust_* = 0.014) and duration (*r* = −0.58, *P_adjust_* = 0.047) of sniffing behavior toward urine from senile males. The relative abundance of *Chryseobacterium* was significantly negatively correlated with the sniffing frequency (*r* = −0.70, *P_adjust_* = 0.011) and duration (*r* = −0.69, *P_adjust_* = 0.011) toward urine from adult males (Figure 6b).

## 4. Discussion

In this study, compared with the DS–pre, females in the DS–post showed increased relative abundances of putatively beneficial bacteria, such as Muribaculaceae and *Weissella cibaria*, and decreased abundances of pathogenic or opportunistic taxa, including Enterobacteriaceae and *Campylobacter*. These results indicate that dried yeast supplementation was associated with shifts in specific gut bacterial taxa in captive female giant pandas, although no detectable changes were observed in overall microbial diversity. In the post-supplementation odor preference test, females showed a significant preference for adult male over senile male urine, as reflected by a higher exploration frequency and longer exploration duration. In addition, Spearman correlation analysis revealed that the relative abundances of gut bacterial genera were significantly associated with exploratory behavior in female giant pandas (Figure S4).

### 4.1. Shifts in Specific Gut Bacterial Taxa Following Dried Yeast Tablet Supplementation in Captive Female Giant Pandas

In the present study, the gut microbiota of captive female giant pandas was dominated by Firmicutes and Proteobacteria, with *Streptococcus*, *Clostridium*, *Turicibacter*, and *Escherichia–Shigella* representing the predominant genera (Figure 2), consistent with previous studies [60,61]. Neither α- nor β-diversity differed significantly after dried yeast tablet supplementation, indicating that the supplement did not result in detectable changes in overall microbial diversity or community-level structure [29,62]. Instead, dried yeast supplementation was associated with taxon-specific changes in the relative abundance of several bacterial taxa (Figure 2). Because giant pandas rely primarily on fibrous bamboo, gut microorganisms may contribute to host physiology by participating in the fermentation of plant-derived carbohydrates and producing metabolites such as short-chain fatty acids [46,48]. Therefore, changes in specific bacterial taxa following dietary supplementation may reflect shifts in microbial fermentation potential and host-associated metabolic processes [63,64]. Specifically, the increased relative abundances of potentially beneficial taxa, including Muribaculaceae and *W. cibaria*, in female giant pandas [65]. Members of the Muribaculaceae are thought to degrade complex carbohydrates and produce short-chain fatty acids and may contribute to intestinal barrier maintenance, immune and inflammatory regulation, and host energy metabolism [66,67]. In mice, changes in Muribaculaceae abundance have been associated with propionate production and increased lifespan [67,68]. *W. cibaria*, a lactic acid bacterium, has been reported to exhibit antimicrobial, antagonistic, and antioxidant activities [69,70]. Moreover, glucans produced by *W. cibaria* may promote the growth of probiotic bacteria, such as *Lactobacillus acidophilus* [71].

Dried yeast tablet supplementation was also associated with decreased relative abundances of *Bergeyella*, *Chryseobacterium*, *Actinomyces*, *Terrisporobacter*, *Lautropia*, *Romboutsia*, *Leucobacter*, and *Campylobacter* (Figure 2). Some members of *Campylobacter*, *Chryseobacterium*, *Actinomyces*, *Terrisporobacter*, and *Bergeyella* have been associated with gastrointestinal disease, opportunistic infections, inflammatory responses, or other adverse health outcomes in their hosts [72,73,74,75,76]. Therefore, the decreased relative abundances of these taxa suggest that dried yeast tablet supplementation may limit the enrichment of potentially harmful bacteria. As a dietary supplement, dried yeast tablets may have potential applications in routine nutritional management and gut health maintenance in captive giant pandas [77]. Yeast and yeast-derived products contain various nutrients and bioactive components, including proteins, B vitamins, beta-glucans, and mannan oligosaccharides [25,26,29]. Previous studies suggest that these components may contribute to host health through multiple mechanisms, including modulation of gut microbial communities, enhancement of intestinal barrier integrity, and regulation of immune responses, and alteration of host metabolism [25,26,29]. Furthermore, direct effects of yeast-derived nutrients or bioactive compounds on host metabolism, immune function, or sensory processes cannot be excluded [78,79]. Therefore, the microbial changes observed in this study may represent one component of broader physiological responses induced by dried yeast supplementation.

### 4.2. Dried Yeast Supplementation May Be Associated with Changes in Female Selectivity Toward Adult Male Urinary Cues

In this study, dried yeast tablet supplementation was associated with altered female exploration response toward urinary odors from adult and senile males (Figure 3 and Figure 4), suggesting that dietary supplementation may influence female behavioral responsiveness to male chemical cues [13,14,31,80]. Before supplementation, females in the DS-treated group showed no significant exploratory preference between urine from adult and older males (Figure 3 and Figure 4). This absence of preference may indicate that urinary odors from both age classes contained sufficient reproductive or age-related cues to elicit female exploration [9,81]. Following supplementation, however, females showed stronger exploratory preference toward adult male urine than toward older male urine (Figure 3 and Figure 4). This shift is consistent with evidence from mice showing that senile males can produce lower concentrations of major urinary proteins and androgen-dependent volatile compounds, which may reduce the attractiveness of their urinary scent signals to females [82]. In addition, female giant pandas showed a shorter latency to first exploration of adult male urine odors after dried yeast tablet supplementation (Figure 5). This result suggests that supplementation was associated with changes in female behavioral responsiveness to urinary odor cues from adult males, although it does not directly demonstrate improved odor recognition or mate preference. Previous studies have reported associations between probiotic, prebiotic, and other dietary treatments and changes in gut microbial communities and host neurobehavioral function [83,84]. However, most available evidence comes from animal models and human clinical or observational studies, and its applicability to giant pandas remains uncertain [83]. Future studies should integrate molecular analyses of odor cues, neuroendocrine measures, stress hormone levels, and reproductive outcomes to clarify whether and how gut microorganisms are linked to olfactory mate-choice behavior in female giant pandas.

In carnivorous mammals, nutritional conditions can constrain reproductive investment, particularly in species with seasonal reproduction and high energetic demands, such as polar bears [85]. Moreover, olfactory communication is a fundamental component of social and reproductive interactions in many carnivores, with females using scent cues to assess potential mates, as reported in pumas and American mink [86,87,88]. Gut microbial composition is also closely linked to dietary ecology and host physiological adaptation across mammals [89,90]. Several bacterial taxa, including *Serratia*, *Carnobacterium*, *Comamonas*, *Psychrobacter*, *Empedobacter*, *Jeotgalibaca*, *Actinomyces*, and *Chryseobacterium,* was associated with female olfactory behavioral variables in the present study. Although none of these taxa have been directly demonstrated to regulate sexual selection or mate preference in carnivores, previous studies suggest that members of these genera participate in diverse physiological processes, including nutrient metabolism, immune regulation, and host–microbe interactions [91,92]. Notably, dried yeast tablet supplementation significantly reduced the relative abundance of the potentially pathogenic genus *Chryseobacterium* in female giant pandas [74]. The relative abundance of *Chryseobacterium* was negatively correlated with both the frequency and duration of female exploration of urine odors from adult males (Figure 6b). These results show that variation in *Chryseobacterium* abundance was associated with differences in female exploration responses toward adult male urine. Although MGBA provides a potential framework linking gut microbial variation with host sensory and behavioral regulation [22,51,93,94], the present study cannot determine whether such pathways contributed to the observe olfactory responses because relevant metabolic and neuroendocrine indicators were not measured. Collectively, dried yeast tablet supplementation was associated with both gut microbial changes and altered behavioral responses to male odor signals. Although these findings are consistent with the possibility that gut microbial variation may contribute to olfactory behavioral regulation, the underlying biological pathways remain to be determined.

## 5. Conclusions

In conclusion, dried yeast tablet supplementation was associated with changes in relative abundance of specific gut bacterial taxa in captive giant panda, while overall microbial diversity and community structure remained unchanged. Behaviorally, supplemented females showed altered olfactory exploration toward male urinary odor, including stronger and more prolonged responses to urine from adult males and a shorter latency to first exploration. Moreover, the relative abundance of *Chryseobacterium* was negatively correlated with female exploration of adult male urine odors, suggesting a potential link between gut microbial composition and mate-choice-related olfactory behavior. Together, these findings suggest that dried yeast supplementation was associated with changes in specific gut bacterial taxa and female olfactory preference for adult male urine in giant pandas. Although the present study does not establish a direct causal mechanism, it provides evidence that gut microorganisms are linked to sexual selection-related behavior in a large mammal and offers a potential microbiota-based perspective for improving reproductive management in captive giant panda populations.

## Data Availability

The nucleotide sequences of bacterial communities were deposited to the NCBI sequence read archive with accession numbers SRR39425386–SRR39425401, SRR39548417–418.

## Supplementary Materials

The following supporting information can be downloaded at https://www.mdpi.com/article/10.3390/ani16162574/s1, Figure S1: α-diversity indices of gut bacterial communities in female giant pandas across the control–pre, control–post, DS–pre, DS–post; Figure S2: Principal component analysis (PCA, a) and non-metric multidimensional scaling (NMDS, b) of gut bacterial community at the genus level in female giant pandas across different groups; Figure S3: Relative abundance of gut microbiota at the phylum (a) and genus (b) levels in female giant pandas of DS- and control groups; Figure S4: Summary diagram illustrating the associations among dried yeast supplementation, gut microbiota, and olfactory behavioral responses in captive female giant panda; Table S1: Subjects information; Table S2: The ethogram and behavioral definitions of female giant pandas.

## Abbreviations

The following abbreviations are used in this manuscript:

DS-treated	Dried yeast-supplemented treated
MGBA	Microbiota–gut–brain axis
CCRCGP	China Conservation and Research Center for the Giant Panda
ARRIVE	Animal Research: Reporting of In Vivo Experiments
ELISA	Enzyme-linked immunosorbent assay
E1G	Estrone-3-glucuronide
Q-PCR	Quantitative real-time polymerase chain reaction
FDR	False discovery rate
ASV	Amplicon sequence variants
