# Supplementary material for: Gut Microbiota Is Linked to Female Olfactory Preference in Giant Pandas
Source: Animals (Basel). 2026 Aug 18;16(16):2574. doi: 10.3390/ani16162574 (PMC13508970; doi:10.3390/ani16162574)
Supplement: Supplementary file 1 [file animals-16-02574-s001.zip › animals-4462372-supplementary.pdf]

Supplementary figures and tables:

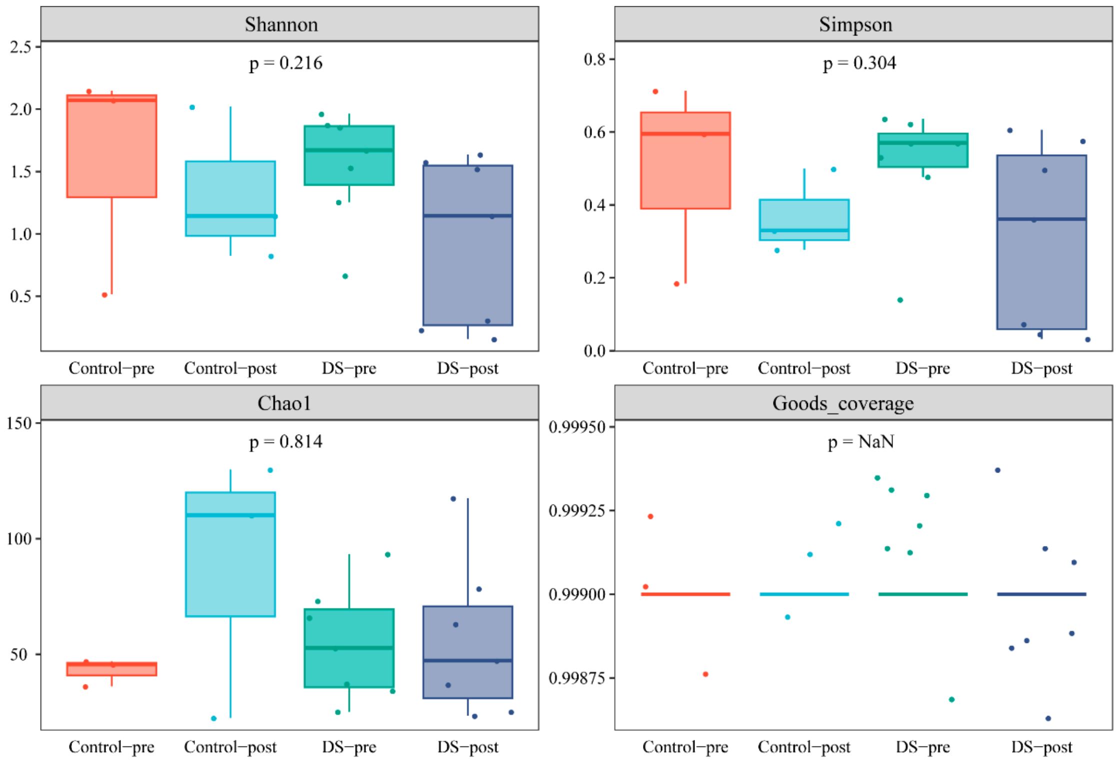

Figure S1 α-diversity indices of gut bacterial communities in female giant pandas across the control–pre, control–post, DS–pre, DS–post.

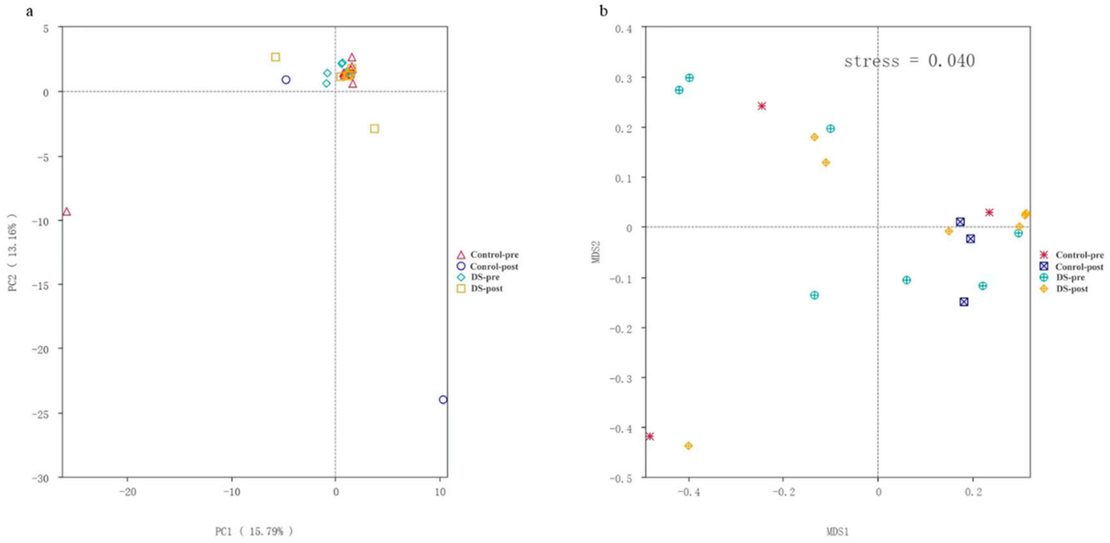

Figure S2 Principal component analysis (PCA, a) and non-metric multidimensional scaling (NMDS, b) of gut bacterial community at the genus level in female giant pandas across different groups.

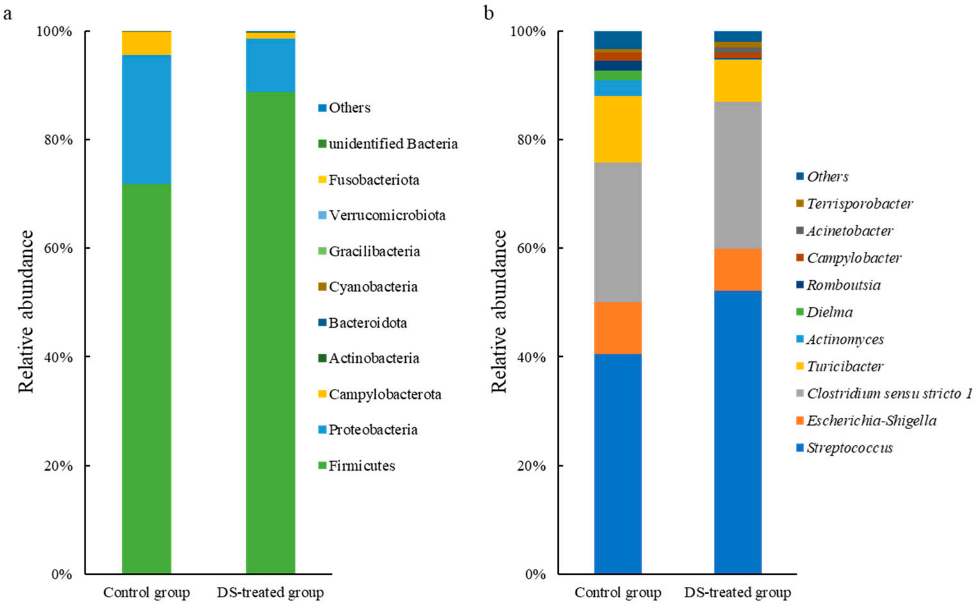

Figure S3 Relative abundance of gut microbiota at the phylum (a) and genus (b) levels in female giant pandas of DS- and control groups.

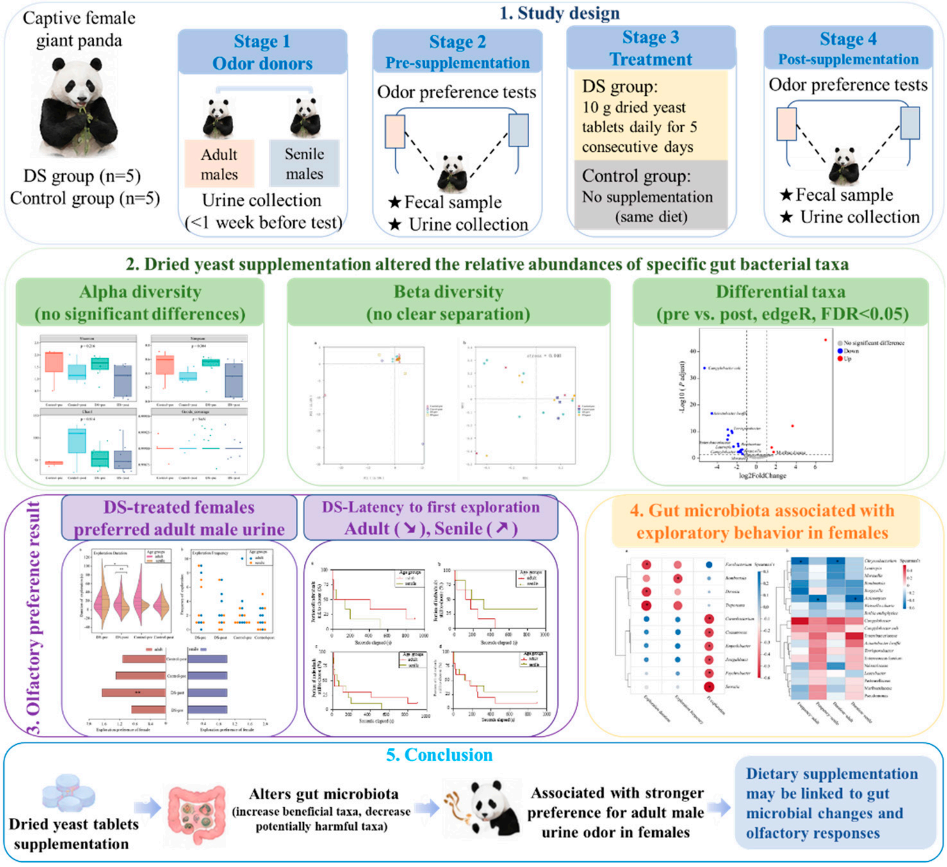

Figure S4 Summary diagram illustrating the associations among dried yeast supplementation, gut microbiota, and olfactory behavioral responses in captive female giant panda.

Table S1 Subjects information

Name	Gender	At the age of 2023	Group
Qiao Yue	Female	5	DS-treated
Pan Yue	Female	7	DS-treated
Yang Hua	Female	13	DS-treated
Min Min	Female	15	DS-treated
Wang Jia	Female	16	DS-treated
Lin Lang	Female	6	Control
Pan Qing	Female	8	Control
Xian Xian	Female	9	Control
Bing Jing	Female	9	Control
Hui Hui	Female	11	Control
Bing Cheng	Male	9	Adult group
Sen Sen	Male	10	Adult group
Hua Hu	Male	10	Adult group
Xiang Lin	Male	14	Adult group
Jin Ke	Male	14	Adult group
Zi Yan	Male	14	Adult group
Ya Xiang	Male	22	Senile group
Lin Yang	Male	22	Senile group
Dai Li	Male	24	Senile group
Wu Gang	Male	24	Senile group
Xi Meng	Male	30	Senile group
Gao Gao	Male	31	Senile group

Table S2 The ethogram and behavioral definitions of female giant pandas

Behavior category	behavior	Definition
Non-contact exploration	Sniffing	Approached the target until its nose was within 5 cm, paused for more than 1 second, focused her eyes on the object, and sniffed with rapid inhalations
	Tail up	Raise tail to expose vulvar to urine carrier.
Contact exploration	Gnaw	Bite the urine carrier with teeth.
	Rubbing	Rub the urine carrier against the body
	Rub anogenital	Place the urine carrier close to the perianal gland area, and rub it in a straight or curved motion, leaving scent marks.
	Bite/grasp the urine carrier	Use the mouth to hold or use the hand to grasp the urine carrier.
	Licking	Lick the urine carrier with the tongue.
